# Vasculitis in Systemic Sclerosis

**DOI:** 10.1155/2010/385938

**Published:** 2010-09-30

**Authors:** Lily Kao, Cornelia Weyand

**Affiliations:** Division of Immunology and Rheumatology, School of Medicine, Stanford University, Stanford, 1000 Welch Road, Suite #203, Palo Alto, CA 94304, USA

## Abstract

Systemic sclerosis (SSc) is a multiorgan connective tissue disease characterized by autoantibody production and fibroproliferative stenosis of the microvasculature. The vascoluopathy associated with SSc is considered to be noninflammatory, yet frank vasculitis can complicate SSc, posing diagnostic and therapeutic challenges. Here, we have reviewed the literature for reports of small-, medium-, and large-vessel vasculitis occurring in SSc. Amongst 88 reported cases of vasculitis in SSc, patients with ANCA-associated vasculitis appear to present a unique subclass in that they combined typical features of SSc with the renal manifestation of ANCA-associated glomerulonephritis. Other vasculitic syndromes, including large-vessel vasculitis, Behcet's disease, cryoglobulinemia, and polyarteritis nodosa, are rarely encountered in SSc patients. ANCA-associated vasculitis needs to be considered as a differential diagnosis in SSc patients presenting with renal insufficiency, as renal manifestations may result from distinct disease processes and require appropriate diagnostic testing and treatment.

## 1. Introduction

Systemic sclerosis (SSc) is a connective tissue disease characterized by fibrosis and vasculopathy involving multiple organ systems. Classification into diffuse or limited cutaneous forms depends on the extent of skin thickening, with the former affecting areas proximal to the elbows or knees, and the latter limited to the face and distal extremities [1]. Many clinical complications of SSc are due to dysfunction of vascular beds throughout the body. Involvement of the microvasculature leads to cutaneous and mucosal telangiectasias, digital ulcers, and tissue ischemia. If medium-sized blood vessels are involved, manifestations include gangrene, digital loss, renal crisis, and pulmonary arterial hypertension [2]. While occlusive vasculopathy is a well-recognized feature of SSc, less is known about the occurrence and the consequences of frank vascular inflammation. Albeit rare, typical vasculitis with inflammatory infiltrates damaging blood vessels has been reported in patients with SSc. Distinguishing between noninflammatory vasculopathy and vasculitis can pose a significant diagnostic challenge in the absence of histologic examination. Here, we review reported cases of large-, medium-, and small-vessel vasculitis in association with SSc.

## 2. Vasculopathy versus Vasculitis

The distinction between SSc vasculopathy and vasculitis can be difficult to make based on clinical presentation alone, but knowledge of the underlying pathogenesis and histopathology can be very helpful. In the current pathogenic model of SSc, a vascular injury of unknown cause leads to endothelial apoptosis and initiates the process of SSc vasculopathy. Autoantibodies, reperfusion injury, infection, and defects in vascular repair have all been implicated as possible instigators [3]. Increased levels of endothelial cells in the circulation have been cited as evidence that the intactness of the vascular lining is jeopardized [3, 4]. Subsequent endothelial dysfunction results in the imbalance of vasoactive factors: decreased levels of vasodilators such as endothelial nitric oxide synthase and prostacyclin synthase, as well as increased levels of the vasoconstrictor endothelin-1 and vascular endothelial growth factor [5, 6]. Continuous endothelial dysfunction likely contributes to activation of adventitial fibroblasts with resultant intimal proliferation, eventual luminal narrowing, and tissue hypoxia [4, 7]. Histopathology of SSc vasculopathy reflects the underlying pathogenesis, with myofibroblast proliferation and matrix deposit in the subendothelial layer leading to obliterative thickening of vessel walls. Inflammatory infiltrates are absent, and the internal elastic lamina remains intact [8]. 

In contrast to vasculopathy, concurrent vasculitis in SSc shows histopathologic evidence of inflammation, with presence of mononuclear infiltrates and destruction of the vascular wall. Notably, both vasculopathic and vasculitic changes were seen in five of nine (55%) digital amputation specimens from SSc patients, emphasizing that small vessel vasculitis and stenosing vasculopathy may coexist [8]. Further support has come from autopsy studies of SSc patients, where 24% of 58 cases showed noninflammatory intimal proliferation in two or more organs, but 9% had features of inflammatory polyarteritis [9]. Thus, vasculitis is known to occur even in the setting of a disease predisposing towards vasculopathy, and histology is required to distinguish the two pathogenic processes.

## 3. Large-Vessel Vasculitis 

### 3.1. Giant Cell Arteritis

Giant cell arteritis is a common vasculitis of the elderly involving large- and medium-sized arteries, typically the temporal, ophthalmic, vertebral, and axillary arteries as well as the aorta. The American College of Rheumatology (ACR) criteria include at least three of the following: (1) onset at age >50, (2) new headache, (3) claudication of the jaw or tongue, (4) temporal artery tenderness to palpation or decreased pulsation, (5) ESR >50 mm/h, and (6) temporal artery biopsy showing vasculitis with mononuclear inflammatory infiltrate or granulomatous inflammation with presence of giant cells [10]. Typical histomorphologic findings include disruption of the internal elastic lamina, thinning of the media, and occlusion of the lumen by hyperplastic intima. Pathogenic studies have established that giant cell arteritis is a T-cell driven disease with participation of Th1 and Th17 lineages [11, 12]. Steroids remain the mainstay of therapy, with many cases resolving after one to two years.

While giant cell arteritis is relatively common among the vasculitides, it has only been reported in three cases of concurrent SSc, all of which were women in their sixth decade with limited skin involvement [13–15] (Table 1). Two of the three had the classic presentation of headache, jaw claudication, and elevated sedimentation rate (ESR), with evidence of vessel wall inflammation and giant cells on temporal artery biopsy. The case reported by Sari-Kouzel was unusual in that the presenting symptom was pain and discoloration of the right foot in the setting of normal ESR. The lower extremity ischemia eventually progressed to gangrene necessitating a below-the-knee amputation. While SSc vasculopathy may have contributed to ischemic tissue damage, the histology from the amputation specimen yielded evidence for vasculitis with the presence of inflammatory infiltrates, giant cells in the vessel wall, and vascular lumen obliteration. All patients were started on corticosteroids (prednisolone or prednisone 30 to 80 mg daily) with slow taper over 5–6 months and resolution of symptoms.

### 3.2. Takayasu Arteritis

Takayasu arteritis is a relatively rare large vessel vasculitis (incidence 0.4–2/million/year) affecting mostly young women of Asian origin although the incidence among the middleaged with atherosclerosis has been rising [20, 21]. The aorta and its main branches are typically involved. ACR diagnostic criteria include at least three of the following: (1) onset before age 40, (2) claudication of an extremity, (3) decreased brachial artery pulse, (4)  >10 mmHg in systolic blood pressure between the arms, (5) bruit over the subclavian arteries or aorta, and (6) stenosis/occlusion of the aorta, its major branches, or large arteries in proximal upper or lower extremities [22]. Similar to giant cell arteritis, the histopathology in Takayasu arteritis shows mononuclear infiltrates in the vessel wall, intimal thickening, destruction of elastic laminas, giant cell formation, and expansion of the adventitial layer. Elastic lamina destruction can lead to aneurysm formation while transmural inflammation drives intimal proliferation, adventitial scarring, and vascular lumen narrowing [23]. Treatment with steroids leads to remission in 40% of patients while 40% may relapse or require addition of a second drug such as methotrexate or azathioprine [24, 25].

Four cases of Takayasu arteritis in the setting of SSc have been reported. As the overwhelming majority of patients with Takaysu arteritis are female, all four of these cases were women, with ages ranging from 29 to 68 [16–19]. Three of the patients had diffuse skin involvement. The presenting symptoms included arm claudication and lightheadedness, and physical examination revealed pulselessness in upper extremities, blood pressure discrepancies  >10 mmHg as measured in both arms, and in one case bruits involving the neck and the back. Computed tomographic angiography in all cases showed stenosis of various aortic branches, including the brachiocephalic trunk, common carotid, subclavian, celiac, and renal arteries. Thoracic outlet syndrome was concurrently diagnosed in one case of arteritis, with imaging demonstrating compression of the brachial artery by the scalenus muscle. Two of the four patients were older than 40 years of age at the time of Takayasu arteritis diagnosis, raising the question whether they indeed had typical large vessel vasculitis or whether a component of vessel-obstructive and progressive atherosclerosis was part of the disease process. The reports did not provide information about therapeutic management. 

## 4. Medium- and Small-Vessel Vasculitis

### 4.1. Polyarteritis Nodosa

Polyarteritis nodosa (PAN) is a necrotizing vasculitis affecting medium-sized vessels, with a constellation of clinical findings that reflect multiorgan involvement. It can be associated with hepatitis B viral infection. PAN can be distinguished from the small-vessel vasculitides such as microscopic polyangiitis by the absence of antineutrophil cytoplasmic antibodies. The ACR diagnostic criteria for PAN include at least three of the following: (1) weight loss >4 kg, (2) livedo reticularis, (3) testicular pain or tenderness, (4) myalgias, weakness, or leg tenderness, (5) mono- or polyneuropathy, (6) hypertension, (7) elevated blood creatinine or urea, (8) serum hepatitis B antigen or antibody, (9) aneurysms or occlusions of visceral arteries, or (10) granulocytes on biopsy of small- or medium-sized arteries [26]. A recent retrospective study of 348 patients with PAN found general symptoms in 93.1%, neurologic involvement in 79%, and skin involvement in about 50% [27]. Five-year relapse-free survival was 59.4% for nonhepatitis-B-associated PAN and 67% for HBV-associated PAN. 

 Predictors of mortality included age greater than 65 years, new onset hypertension, and gastrointestinal manifestations requiring surgery. Treatment relies on glucocorticoids in mild disease and a combination of glucocorticoids and cyclophosphamide in moderate to severe disease.

Only one case of PAN has been described in a 28-year-old woman with diffuse SSc, characterized by Raynaud's phenomenon and skin sclerosis over the hands, arms, and chest [28] (Table 2). The patient developed brownish tender nodules on her legs over three months. Biopsy of these lesions revealed necrotizing arteritis in the deep dermis. Despite the histologic appearance of the nodules, the patient technically did not meet ACR criteria for PAN as she had normal blood pressure and renal function, negative hepatitis B serologies, and no other symptoms such as weakness or neuropathy. Symptoms responded to weekly methotrexate at 20 mg and remained stable at five months.

### 4.2. Primary Angiitis of the Central Nervous System

Primary angiitis of the central nervous system (PACNS) is a rare poorly characterized entity affecting small- and medium-sized vessels of the central nervous system (CNS) but not organs or vessels outside the CNS. In general, PACNS is distinguished from secondary CNS vasculitis with the exclusion of infections, malignancy, systemic vasculitis or connective tissue disease, or drug-induced vasculitis. Clinical presentations of PACNS include confusion, new onset headache, seizures, stroke or cerebral hemorrhage, and myelopathy [35]. The duration from symptom onset to diagnosis can range from 3 days to 3 years [36]. Multiple laboratory data abnormalities can occur but none is specific for the diagnosis, with ESR described to be normal in a number of cases. Characteristic changes on cerebral angiography include multifocal segmental stenosis, dilatation, or occlusion of small- and medium-sized leptomeningial and intracranial vessels as well as formation of collateral vessels. Further supportive evidence can be obtained from leptomeningeal or parenchymal biopsies, which are specific but not sensitive for vasculitis given the focal segmental nature of the disease; therefore, a negative biopsy does not rule out the diagnosis. Histology can show either granulomas in small vessel walls, lymphocytic infiltrates, or necrotizing vasculitis [36]. The rarity and heterogeneity complicate classification, diagnosis, and management. Calabrese and Mallek have proposed the following diagnostic criteria for PACNS: (1) recent onset of headache, confusion, or multifocal neurologic deficits, (2) cerebral angiographic changes suggestive of vasculitis, (3) exclusion of systemic disease or infection, and (4) leptomeningeal or parenchymal biopsy to confirm vasculitis and to exclude infection, malignancy, and noninflammatory vascular occlusive disease [37]. Once the diagnosis is made, aggressive treatment with high-dose steroids and cyclophosphamide has been suggested as the management of choice.

One case of PACNS has been described in SSc [29] (Table 2). A 45-year-old woman with limited SSc diagnosed at age 21 presented with new onset headache for 3 days and confusion, later developing hypertension to the 230s/190s and generalized seizure. Computed tomography of the head and spinal fluid was unremarkable. Cerebral angiogram showed an occluded medium-sized branch of the middle cerebral artery as well as narrowing of several distal medium-sized arteries in the anterior and middle cerebral artery distribution. The patient was empirically started on methylprednisolone 100 mg IV every 4 hours for suspicion of PACNS, and her mental status was normalized within 14 hours. A repeat cerebral angiogram of the posterior circulation showed multiple 1.5 cm segments of smooth narrowing in medium-sized arteries. The steroid dosage could not be tapered below prednisone 50 mg daily, as each time the patient developed right facial and arm paresthesias and expressive aphasia. Despite a negative leptomeningeal biopsy, cyclophosphamide was started at 100 mg daily and gradually increased to 200 mg daily with complete resolution of symptoms.

### 4.3. Mixed Cryoglobulinemia and Cryofibrinogenemia

Mixed cryoglobulinemia is the presence of polyclonal immunoglobulins that precipitate in the serum with cold exposure, often secondary to a connective tissue disease such as systemic lupus erythematosus or Sjogren's syndrome. The presence of cryoglobulins (CGs) may be asymptomatic or may lead to manifestations of the cryoglobulinemic syndrome, including purpura, arthralgia, myalgia, glomerulonephritis, and peripheral neuropathy [38]. The diagnosis of the latter entails a combination of clinical presentation, laboratory testing showing the presence of circulating cryoglobulins, and histopathologic appearance such as leukocytoclastic vasculitis (most common). Similarly, cryofibrinogenemia is the presence of cold-induced precipitants in the plasma but not in the serum. Connective tissue diseases, malignancy, and infection have been known to be associated with this condition, which can be asymptomatic or can manifest as painful ulcers, purpura, or perniosis, reflecting possible underlying cold-induced thromboses, increased blood viscosity, or vascular reactivity. The diagnosis of clinically significant cryofibrinogenemia requires not only circulating cryofibrinogen (CF) but also clinical features and histopathologic evidence of small-vessel thrombosis and perivascular infiltrate [39]. For both mixed cryoglobulinemia and cryofibrinogenemia, treating the underlying disease (whether infection, connective tissue disease, or malignancy) can sometimes improve symptoms. Plasmapheresis and immunosuppression with glucocorticoids and/or cytotoxic therapy have also been used in severe disease although with unclear efficacy.

Connective tissue diseases have been associated with the presence of both CG and CF, perhaps more so than CF alone [40]. In the few studies and reports involving SSc, these cold-induced precipitants do not appear to trigger symptoms. In one study, one out of 19 patients with both CG and CF carried the diagnosis of SSc [40]. In another study, 10 out of 20 SSc patients had the presence of polyclonal IgG and IgM cryoglobulins in the serum, but none exhibited clinical signs of cryoglobulinemic syndrome [41]. In one report of long-standing SSc with the presence of cryoglobulins (both IgG and IgM), the patient presented with paresthesias, transient aphasia, vision changes, and delirium [30] (Table 2). Cerebral angiogram was normal, and electroencephalogram revealed generalized slowing of action potentials, and computed tomography of the extremities revealed calcinosis. While peripheral neuropathy can be a manifestation of mixed cryoglobulinemia, central nervous system involvement would be highly unusual; therefore it, is unclear whether the presence of cryoglobulins in this case is an incidental finding. Another man with long-standing SSc presented with sudden onset gangrene in the fingers and toes after cold exposure and was found to have very elevated cryofibrinogen [31]. He did not respond to prostaglandin E1 or subcutaneous heparin and died shortly after presentation. No biopsy was done to ascertain the etiology for the gangrene, therefore, either underlying SSc vasculopathy or thrombosis from the cryofibrinogenemia could have been possible causes. 

### 4.4. Behcet's Disease

Behcet's disease is characterized by recurrent oral aphthous ulcers and other systemic manifestations believed to be due to systemic vasculitis, including genital aphthous ulcers, ocular disease, skin involvement, gastrointestinal ulcers, neurologic disease, and arthritis. It is more common along the ancient Silk Road, with 0.11% prevalence in Turkey and 2.6% per 100,000 in Southern China [42]. Diagnosis is made based on clinical features including presence of recurrent oral aphthae plus two of the following without other systemic disease: (1) recurrent genital aphthae, (2) eye lesions including uveitis or retinal vasculitis, (3) skin lesions including erythema nodosum, pseudovasculitis, papulopustular lesions, or acneiform nodules, or (4) positive pathergy test [43]. Treatment for mucocutaneous and joint disease includes colchicine (mixed results), glucocorticoids, and other immunosuppressives such as azathioprine. More serious disease with internal organ involvement has been treated with cyclophosphamide and high-dose steroids.

Two cases of Behcet's disease with concurrent SSc have been reported [32, 33] (Table 2). Recurrent oral and genital ulcers were the predominant symptoms in both cases, and neither had eye involvement. In the case of the 54-year-old woman with diffuse SSc, the diagnosis of Behcet's disease was made based on recurrent oral and genital ulcers and presence of pathergy; skin involvement was nonspecific [32]. Her more bothersome symptoms were related to her SSc including restrictive lung disease, esophageal dysmotility, and polyarthritis, as well as secondary Sjogren's syndrome. The recurrent ulcers initially responded well to azathioprine 100 mg daily; later she was switched to colchicine with unknown effect. In the case of the 62-year-old man with limited SSc based on biopsy-proven sclerodactyly, the presence of recurrent oral and genital ulcers as well as erythema nodosa led to the diagnosis of Behcet's disease [33]. He had concurrent chronic hepatitis C complicated by pancytopenia. He later developed esophageal and gastric ulcers that were felt to be consistent with enteric Behcet's. These ulcers were resistant to prednisolone 30 mg daily.

### 4.5. Relapsing Polychondritis

Relapsing polychondritis (RPC) is an inflammatory disease of unknown etiology involving cartilagenous tissues in multiple organs, typically the ears, nose, eyes, respiratory tract, and joints. Vascular and neurologic complications have also been reported. Association with systemic vasculitis, connective tissue disease, or myelodysplastic syndrome occurs in up to one-third of the cases. The original diagnostic criteria by McAdam required three of six clinical manifestations: (1) bilateral auricular chondritis, (2) nonerosive seronegative polyarthritis, (3) nasal chondritis, (4) respiratory tract chondritis, (5) ocular inflammation, or (6) cochlear and/or vestibular dysfunction [44]. The criteria were later modified to include the presence of three or more of the above, one clinical manifestation with corroborating histology, or chondritis at more than two sites responsive to steroids or Dapsone [45]. Given the rarity and relapsing nature of the disease, treatment has been empiric, with Dapsone and glucocorticoids favored for mild nonvisceral disease, and high-dose steroids and possible adjunctive immunosuppressants for organ-threatening disease.

Only one case of RPC has been reported in association with SSc [34] (Table 2). A 35-year-old man with limited SSc presented with a known nasal septal perforation presented with left auricular swelling and polyarthralgia. The left auricle later was ulcerated with drainage of pus and was felt to be super infected with *Pseudomonas aeruginosa*. However, the ulcer did not improve with antibiotics but rather with prednisolone 15 mg daily.

## 5. ANCA-Associated Vasculitis (AAV)

The spectrum of necrotizing small-vessel vasculitis known as ANCA-associated vasculitis (AAV) includes Wegener's granulomatosis (WG), microscopic polyangiitis (MPA), and Churg-Strauss syndrome (CSS). While formally classified as small-vessel vasculitis, AAV can involve medium-sized vessels. Cytoplasmic antineutrophil cytoplasmic antibodies (c-ANCAs) directed against proteinase 3 (PR3) are more commonly found in WGs whereas perinuclear ANCAs (p-ANCA) targeting myeloperoxidase (MPO) are more frequently seen in MPA and CSS. Variable organ involvement makes diagnosis a challenge, with alveolar hemorrhage and crescentic glomerulonephritis frequently occurring in WG and MPA, while polyneuropathy can be seen in ANCA-associated CSS [46]. Disease stage can range from localized without end-organ damage to severe generalized with organ failure. Treatment of generalized disease involves induction with cyclophosphamide (whether oral or intravenous) followed by maintenance with less toxic drugs such as azathioprine, methotrexate, or myclophenolate mofetil. Standard therapy leads to remission in 90% to 94% of AAV patients within six months although relapses frequently occur (18 to 40% in WG at 24 months) [47].

Of all the small vessel vasculitides, AAV is the most frequently reported in association with SSc, raising the question whether an overlap syndrome exists that combines features from both diseases. A study by Rho et al. found 31 reports containing 63 cases of AAV in SSc up to 1994 [56]. Fifty of the 63 cases provided sufficient clinical and laboratory information and were included in the analysis. Eighty-four percent were women with a mean age of 57.1 years. Cases of limited and diffuse SSc were equally represented. Autoantibody profiling showed 72% with positive ANA (titers unknown), 70% with anti-Scl-70 antibody, and 72% with positive anti-MPO antibody. The most common end-organ involvement included kidneys (82%) and lungs (70% had pulmonary fibrosis). Outcomes were not described in 17/50 (34%), but analysis of the remaining cases yielded a 7-year survival rate of 67.9%, with a high mortality rate within the first year. Furthermore, anti-Scl-70 antibody was found to be a significant predictor of developing AAV in SSc (OR 3.1, 95% confidence interval 1.11-8.55, *P* value  .031). There was no difference in AAV occurrence between SSc subtypes (*P* value  .998), the presence of MPO versus. PR3 ANCA (*P* value  .196), or prior use of D-penicillamine (*P* value  .143).

In our review of the literature, we found eleven additional cases of AAV in SSc that were not cited as part of the Rho study [48–55] (Tables 3 and 4). Seven (64%) were female with a mean age of 53 ± 14 years (range from 19 to 71 years). SSc disease duration was known for 9 of the 11 cases, with a mean of 4.49 ± 3.37 years (range from 0.42 to 10 years). The time from SSc diagnosis to AAV onset was known for 10 cases and reached a mean of 3.85 ± 2.67 years (range from 0.42 to 8 yrs). Seven patients (64%) had diffuse skin involvement, and four (36%) were previously treated with D-penicillamine. Renal involvement in the form of crescentic glomerulonephritis on renal biopsy was seen in 9 cases (82%) while pulmonary fibrosis was seen in 3 cases (27%). One case was complicated by multiple cerebral hemorrhages [55]. Eight cases (88%) had positive ANA of at least 1 : 320, 5 cases (45%) with positive anti-Scl-70 antibody, and only 1 case (9%) with Anticentromere antibody. Almost all cases (91%) had positive anti-MPO antibody while none had anti-PR3 antibody.

These findings highlight the importance of considering crescentic glomerulonephritis related to AAV as a potential cause of renal insufficiency in SSc patients. Classically, scleroderma renal crisis occurs in up to 20% of patients with diffuse SSc, and renal involvement manifesting as hypertension, proteinuria, or azotemia can be found in 45–60% [57]. However, causes other than scleroderma renal crisis should be considered as a differential diagnosis, especially in settings of normotension or ANCA positivity.

## 6. Conclusion

While lumen-occlusive vasculopathy is a prominent feature of SSc, frank vasculitis may also occur. Coexistent SSc and vasculitis have been reported for vessels of all sizes, either before or after SSc diagnosis, and in either SSc subtype (limited or diffuse). We found in the literature 88 cases of vasculitis in SSc, with the vast majority being ANCA-associated vasculitides (74 cases: 63 cited by previous studies, 11 new). Rare cases of Takayasu's arteritis (4 cases), giant cell arteritis (3 cases), and Behcet's disease (2 cases) have been reported in patients affected by SSc. Only single-case reports have focused on other vasculitic syndromes, including mixed cryoglobulinemia/cryofibrinogenemia, polyarteritis nodosa, primary angiitis of the central nervous system, and relapsing polychondritis, suggesting that the association between SSc and these disorders may be a chance event. Although most patients exhibited classic symptoms and signs for the respective vasculitides, confirmation of diagnoses and distinction from SSc rested on histology. Prompt treatment with immunosuppression usually resulted in stabilization of symptoms. In SSc patients with renal insufficiency and ANCA positivity, crescentic glomerulonephritis related to AAV should be considered as a differential diagnosis.

## Figures and Tables

**Table 1:** Seven cases of large-vessel vasculitis associated with SSc.

Case	Vasculitis	Age/Sex	Age at SSc Dx	SSc type	Age at Vasculitis Dx	Presentation	Diagnosis	Outcome
Perez-Jimenez et al. [13]	GCA	68/F	68	L	70	Headache, jaw claudication; ESR 53	Temporal artery biopsy: inflammatory infiltrate, giant cells	Prednisone 60 mg daily x 5 weeks then tapered. Symptom-free at 5 years

Hupp [14]	GCA	70/F	?	L	70	Headache, jaw claudication; ESR 53	Temporal artery biopsy: mononuclear infiltrate, giant cells, destruction of internal elastic lamina	Prednisone 80 mg daily, tapered to 30 mg daily over 5 months. Symptom-free and ESR 3 at 5 months.

Sari-Kouzel et al. [15]	GCA	64/F	49	L	64	Gangrene of right 2nd and 3rd toes; ESR 14	Right knee amputation biopsy: mononuclear infiltrate, giant cells, vascular lumen occlusion	Prednisolone 30 mg daily, tapered to 10 mg daily over 6 months. Symptom-free at 6 months.

Passiu et al. [16]	TA	29/F	29	D	21	?	?	?

Yago et al. [17]	TA	68/F	66	D	66	Vertigo, bruits in neck, abdomen, and back; asymmetric blood pressure in arms	Stenosis of right brachiocephalic trunk, celiac and left renal arteries	?

Kocabay et al. [18]	TA	48/F	48	D	47	Pulseless in both arms with intermittent claudication	Obliteration of bilateral subclavian arteries distal to vertebral artery bifurcation	?

Kim et al. [19]	TA	37/F	33	L	37	Weak left radial pulse	Stenosis of right common carotid artery, right external carotid artery, thoracic and abdominal aorta, left brachial artery compression by scalenus muscle on abduction	?

GCA = giant cell arteritis; TA = Takayasu's arteritis; SSc = systemic sclerosis; L = limited; D = diffuse; ESR = erythrocyte sedimentation rate.

**Table 2:** Seven cases of medium- and-small vessel vasculitis associated with SSc.

Case	Vasculitis	Age/Sex	Ag at SSc Dx	SSc type	Age at Vasculitis Dx	Presentation	Diagnosis	Outcome
Pathak and Gobor [29]	CNS	45/F	21	L	45	Headache, confusion, seizure, aphasia; right face/arm/leg numbness	Angiography with abrupt cutoff in MCA branch, narrowing in ACA and MCA branches; leptomeningeal biopsy negative	Improved with methylprednisolone then cyclophosphamide

Kang et al. [28]	PAN	28/F	28	D	28	Brownish tender nodules on legs	Skin biopsy: necrotizing arteritis in deep dermis	Stable on weekly methotrexate 20 mg

Jiménez López et al. [30]	Mixed CG	50/F	50	L	50	Paresthesias, vision changes, aphasia, delirium; rash on lips, palms, soles	Cryoglobulin >0.1mg/100mL Electroencephalogram: generalized slowing Cerebral angiogram: normal	?

Barrett et al. [31]	CF	49/M	38	L	49	Sudden onset gangrene of fingers and toes; confusion	Cryofibrinogen 435 mg/L (<40)	Died despite Subcutaneous heparin and prostaglandin E1

Choy et al. [32]	BD	54/F	54	D	16	Oral/genital ulcers, superficial thrombophlebitis, rash, esophageal dysmotility, no tear production, left knee arthritis	Grade II esophagitis ILD with restrictive PFTs Rash with pathergy	Unresponsive to topical steroids; later azathioprine 100 mg daily, eye drops, D-penicillamine, colchicine; arthritis continued

Yokota et al. [33]	BD	62/M	57	L	60	Oral/genital ulcers, erythema nodosa, esophageal and gastric ulcers, chronic hepatitis C, pancytopenia	?	Esophageal ulcers resistant to prednisolone 30 mg daily; died from pneumonia

Sugisaki et al. [34]	RPC	35/M	31	L	35	Left auricular ulcer and swelling; polyarthralgia	MRI: partial defect in nasal septum	Improved with prednisolone 15 mg daily and not with antibiotics

CNS = central nervous system vasculitis; PAN = polyarteritis nodosa; Mixed CG = mixed cryoglobulinemia; CF = cryofibrinogenemia; BD = Behcet's disease; RPC = relapsing polychondritis; SSc = systemic sclerosis; L = limited; D = diffuse.

**Table 3:** Eleven new cases of ANCA-associated vasculitis in SSc.

Case	Age/Sex	SSc (yrs)	SSc to AAV (yrs)	SSc type	Use of D-penicillamine	Renal	Pulm fibrosis	ANA	Scl-70	MPO
Marlier et al. [48]	62/M	5	5	L	Yes	CGN	No	?	?	+
Martínez Ara et al. [49]	63/F	2	2	?	No	CGN	No	1 : 640	+	+
Herrera-Esparza et al. [50]	60/M	2	3	L	No	CGN	No	1 : 2560	?	+
Hidalgo-Tenorio et al. [51]	48/F	1	1.5	D	No	None	Yes	1 : 320	Neg	+
	71/F	?	?	L	No	None	No	1 : 320	Neg	+
Kamen et al. [52]	45/M	0.42	0.42	D	No	CGN	No	1 : 2560	+	+
	19/M	5	5	D	Yes	CGN	No	1 : 2560	+	+
	60/F	8	8	D	No	CGN	No	?	+	+
Arnaud et al. [53]	46/F	10	5.75	D	Yes	CGN	Yes	>1 : 1000	+	+
Ramaswami et al. [54]	52/F	?	0.83	D	No	CGN	No	1 : 640	?	?
Veetil and Schimmer [55]	62/F	7	7	D	Yes	CGN	No	?	?	+

Note: All but Ramaswami had positive MPO, and none had PR3. SSc = Systemic sclerosis; L = Limited; D = Diffuse; CGN = crescentic glomerulonephritis; ? = unknown.

**Table 4:** Characteristics of eleven cases of AAV in SSc.

Characteristic	Number (%)
Sex (M/F)	4/7 (36%/64%)
Age (mean ± SD)	53 ± 14 (range from 19 to 71)
SSc duration (mean ± SD in yrs); 9 pts	4.49 ± 3.37 (range from 0.42 to 10 yrs)
SSc Dx to AAV dx (mean ± SD in yrs); 10 pts	3.85 ± 2.67 (range from 0.42 to 8 yrs)
SSc type (Diffuse/Limited)	7/3 (64%/27%)
D-penicillamine use	4 (36%)
Renal involvement	9 (82%) all with crescentic GN on Bx
Pulmonary involvement	2 (18%)
ANA + (>1 : 320)	8 (88%)
Scl-70 +	5 (45%)
Anticentromere +	1 (9%)
MPO +	10 (91%)
PR3 +	0

## References

[1] Medsger TA (2003). Natural history of systemic sclerosis and the assessment of disease activity, severity, functional status, and psychologic well-being. *Rheumatic Disease Clinics of North America*.

[2] Ebert EC (2008). Gastric and enteric involvement in progressive systemic sclerosis. *Journal of Clinical Gastroenterology*.

[3] Kahaleh B (2008). Vascular disease in scleroderma: mechanisms of vascular injury. *Rheumatic Disease Clinics of North America*.

[4] Fleming JN, Nash RA, Mahoney WM, Schwartz SM (2009). Is scleroderma a vasculopathy?. *Current Rheumatology Reports*.

[5] Müller-Ladner U, Distler O, Ibba-Manneschi L, Neumann E, Gay S (2009). Mechanisms of vascular damage in systemic sclerosis. *Autoimmunity*.

[6] Mulligan-Kehoe MJ, Simons M (2008). Vascular disease in scleroderma: angiogenesis and vascular repair. *Rheumatic Disease Clinics of North America*.

[7] Wigley FM (2009). Vascular disease in scleroderma. *Clinical Reviews in Allergy and Immunology*.

[8] Herrick AL, Oogarah PK, Freemont AJ, Marcuson R, Jayson MIV (1994). Vasculitis in patients with systemic sclerosis and severe digital ischaemia requiring amputation. *Annals of the Rheumatic Diseases*.

[9] D’Angelo WA, Fries JF, Masi. AT, Shulman LE (1969). Pathologic observations in systemic sclerosis (scleroderma). A study of fifty-eight autopsy cases and fifty-eight matched controls. *The American Journal of Medicine*.

[10] Hunder GG, Bloch DA, Michel BA (1990). The American College of Rheumatology 1990 criteria for the classification of giant cell arteritis. *Arthritis and Rheumatism*.

[11] Weyand CM, Goronzy JJ (2003). Medium- and large-vessel vasculitis. *The New England Journal of Medicine*.

[12] Deng J, Younge BR, Olshen RA, Goronzy JJ, Weyand CM (2010). Th17 and th1 T-cell responses in giant cell arteritis. *Circulation*.

[13] Perez-Jimenez F, Lopez-Rubio F, Cañadillas F, Jimenez-Alonso J, Jimenez-Pereperez J (1982). Giant cell arteritis associated with progressive systemic sclerosis. *Arthritis and Rheumatism*.

[14] Hupp SL (1989). Giant cell arteritis associated with progressive systemic sclerosis. *Journal of Clinical Neuro-Ophthalmology*.

[15] Sari-Kouzel H, Herrick AL, Freemont AJ, Marcuson RW, Jayson MI (1999). Giant cell arteritis in a patient with limited cutaneous systemic sclerosis. *Rheumatology*.

[16] Passiu G, Vacca A, Sanna G (1999). Takayasu’s arteritis overlapping with systemic sclerosis. *Clinical and Experimental Rheumatology*.

[17] Yago T, Ota S, Nishinarita M (2002). A case of systemic sclerosis complicated by Takayasu’s arteritis. *Ryumachi*.

[18] Kocabay G, Tiryaki B, Ekmekçi A, Inanç M (2006). Takayasu arteritis associated with systemic sclerosis. *Modern Rheumatology*.

[19] Kim T-J, Uhm W-S, Song S-Y, Jun J-B (2007). Unilateral weak radial pulse in a patient with systemic sclerosis: Takayasu’s arteritis or thoracic outlet syndrome?. *Rheumatology International*.

[20] Reinhold-Keller E, Herlyn K, Wagner-Bastmeyer R, Gross WL (2005). Stable incidence of primary systemic vasculitides over five years: results from the German vasculitis resister. *Arthritis Care and Research*.

[21] Seyahi E, Ugurlu S, Cumali R (2006). Atherosclerosis in Takayasu arteritis. *Annals of the Rheumatic Diseases*.

[22] Arend WP, Michel BA, Bloch DA (1990). The American College of Rheumatology 1990 criteria for the classification of Takayasu arteritis. *Arthritis and Rheumatism*.

[23] Fassbender HG (2002). *Pathology and Pathobiology of Rheumatic Diseases*.

[24] Kerr GS, Hallahan CW, Giordano J (1994). Takayasu arteritis. *Annals of Internal Medicine*.

[25] Hoffman GS (1995). Treatment of resistant Takayasu’s arteritis. *Rheumatic Disease Clinics of North America*.

[26] Lightfoot RW, Michel BA, Bloch DA (1990). The American College of Rheumatology 1990 criteria for the classification of polyarteritis nodosa. *Arthritis and Rheumatism*.

[27] Pagnoux C, Seror R, Henegar C (2010). Clinical features and outcomes in 348 patients with polyarteritis nodosa. *Arthritis and Rheumatism*.

[28] Kang MS, Park JH, Lee CW (2008). A case of overlap between systemic sclerosis and cutaneous polyarteritis nodosa. *Clinical and Experimental Dermatology*.

[29] Pathak R, Gabor AJ (1991). Scleroderma and central nervous system vasculitis. *Stroke*.

[30] Jiménez López A, García Marcos MA, Fernández-Roldán CC, Fuertes Martín K, Hernández Vicente I, Pastor Encinas I (1980). Sindrome CRST y crioglobulinemia mixta. *Medicina Clinica*.

[31] Barrett MC, Prendiville JS, Pardy BJ, Cream JJ (1986). Cryofibrinogenaemia and acute gangrene in systemic sclerosis. *Postgraduate Medical Journal*.

[32] Choy E, Kingsley G, Panayi G (1993). Systemic sclerosis occurring in a patient with Adamantiades-Behcet’s disease. *British Journal of Rheumatology*.

[33] Yokota K, Hirano M, Akiba H (2004). A case of Behcet’s disease with esophageal ulcers complicated with systemic sclerosis, chronic hepatitis C, and pancytopenia. *Japanese Journal of Clinical Immunology*.

[34] Sugisaki K, Takeda I, Kanno T, Kasukawa R (2002). A case report of relapsing polychondritis with an auricular ulcer complicated by systemic sclerosis. *Ryumachi*.

[35] Calabrese LH, Duna GF, Lie JT (1997). Vasculitis in the central nervous system. *Arthritis and Rheumatism*.

[36] Lie JT (1992). Primary (granulomatous) angiitis of the central nervous system: a clinicopathologic analysis of 15 new cases and a review of the literature. *Human Pathology*.

[37] Calabrese LH, Mallek JA (1988). Primary angiitis of the central nervous system. Report of 8 new cases, review of the literature, and proposal for diagnostic criteria. *Medicine*.

[38] Brouet JC, Clauvel JP, Danon F (1974). Biologic and clinical significance of cryoglobulins. A report of 86 cases. *American Journal of Medicine*.

[39] Jantunen E, Soppi E, Neittaanmaki H, Lahtinen R (1993). Essential cryofibrinogenaemia, leukocytoclastic vasculitis and chronic purpura. *Journal of Internal Medicine*.

[40] Blain H, Cacoub P, Musset L (2000). Cryofibrinogenaemia: a study of 49 patients. *Clinical and Experimental Immunology*.

[41] Husson JM, Druet P, Contet A (1976). Systemic sclerosis and cryoglobulinemia. *Clinical Immunology and Immunopathology*.

[42] Yurdakul S, Hamuryudan V, Yazici H (2004). Behçet syndrome. *Current Opinion in Rheumatology*.

[43] Wechsler B, Davatchi F, Mizushima Y (1990). Criteria for diagnosis of Behcet’s disease. *The Lancet*.

[44] McAdam LP, O’Hanlan MA, Bluestone RCM (1976). Relapsing polychondritis: prospective study of 23 patients and a review of the literature. *Medicine*.

[45] Damiani JM, Levine HL (1979). Relapsing polychondritis. Report of ten cases. *Laryngoscope*.

[46] Holle JU, Gross WL (2009). ANCA-associated vasculitides: pathogenetic aspects and current evidence-based therapy. *Journal of Autoimmunity*.

[47] Mukhtyar C, Flossmann O, Hellmich B (2008). Outcomes from studies of antineutrophil cytoplasm antibody associated vasculitis: a systematic review by the European League Against Rheumatism systemic vasculitis task force. *Annals of the Rheumatic Diseases*.

[48] Marlier S, Gisserot O, Yao N (1999). Extracapillary glomerulonephritis in a patient treated with D-peniccillamine. *Presse Medicale*.

[49] Martínez Ara J, Picazo ML, Torre A, Pascual D, Díaz Rodríguez C, Riñón C (2000). Progressive systemic sclerosis associated with anti-myeloperoxidase ANCA vasculitis with renal and cutaneous involvement. *Nefrologia*.

[50] Herrera-Esparza R, Aguilar J-L, Saucedo A, González I, López-Robles E, Avalos-Díaz E (2005). Scleroderma with type III glomerulonephritis and MPO-ANCA antibodies in the serum. *Journal of the European Academy of Dermatology and Venereology*.

[51] Hidalgo-Tenorio C, Càliz R, Gallego M, Jaimez L, Jiménez-Alonzo J (2005). Systemic sclerosis, pulmonary fibrosis and anti-MPO antibodies. *Clinical Rheumatology*.

[52] Kamen DL, Wigley FM, Brown AN (2006). Antineutrophil cytoplasmic antibody-positive crescentic glomerulonephritis in scleroderma—a different kind of renal crisis. *Journal of Rheumatology*.

[53] Arnaud L, Huart A, Plaisier E (2007). ANCA-related crescentic glomerulonephritis in systemic sclerosis: revisiting the “normotensive scleroderma renal crisis”. *Clinical Nephrology*.

[54] Ramaswami A, Kandaswamy T, Rajendran T (2008). Scleroderma with crescentic glomerulonephritis: a case report. *Journal of Medical Case Reports*.

[55] Veetil BMA, Schimmer BM (2009). A case of limited systemic sclerosis with p-ANCA, complicated by multiple cerebral hemorrhages. *Rheumatology International*.

[56] Rho YH, Choi SJ, Lee YH, Ji JD, Song GG (2006). Scleroderma associated with ANCA-associated vasculitis. *Rheumatology International*.

[57] Steen VD, Syzd A, Johnson JP, Greenberg A, Medsger TA (2005). Kidney disease other than renal crisis in patients with diffuse scleroderma. *Journal of Rheumatology*.

